# Individual decision making about lung cancer screening: A conjoint analysis of perspectives among a high‐risk national sample

**DOI:** 10.1002/cam4.2445

**Published:** 2019-08-06

**Authors:** Margaret M. Byrne, Richard J. Thurer, Jamie L. Studts

**Affiliations:** ^1^ Department of Health Outcomes and Behavior H Lee Moffitt Cancer Center Tampa Florida; ^2^ Department of Surgery and Sylvester Comprehensive Cancer Center, Miller School of Medicine University of Miami Miami Florida; ^3^ Department of Behavioral Science, College of Medicine University of Kentucky Lexington Kentucky; ^4^ Cancer Prevention and Control Program University of Kentucky Markey Cancer Center Lexington Kentucky

**Keywords:** decision making, lung cancer screening, patient preferences

## Abstract

**Objectives:**

Lung cancer screening (LCS) is effective in reducing lung cancer mortality, but there is limited information available regarding preferences among high‐risk individuals concerning LCS. In this study, we use a conjoint valuation analysis (CVA) to better understand which LCS attributes most affect LCS preferences.

**Materials and Methods:**

We implemented a web‐based nationally representative survey that included a full‐profile CVA exercise. Participants were over the age of 45, had at least a 20 pack‐year smoking history, and no history of lung cancer. The CVA instrument included five LCS attributes, and additional survey items collected demographic and psychosocial information.

**Results:**

Participants (n = 210) had a mean age of 61 (SD 8.5) years, approximately half were female (51.9%), and were racially/ethnically diverse. Average relative importance of the LCS program attributes was (from high to low): out of pocket costs (27.3 ± 17.7); provider recommendation (24.8 ± 13.4); mortality reduction (17.2 ± 8.9); false‐positive rate (15.8 ± 10.4); and ease of access (14.8 ± 7.3). There was large variation among individuals, but few significant associations of propensity to screen with individual demographic characteristics. Average screening propensity across individuals (1‐9 scale) was 3.63 ± 1.6, and average rates of individual scenarios ranged from 2.60 ± 2.00 to 5.57 ± 2.13, indicating low inclination for screening.

**Conclusions:**

We found that overall propensity for screening is low in a high‐risk population, and that out of pocket costs were of greater importance to potential screeners than mortality reduction or false‐positive rates. Thus, individuals considering or eligible for LCS need additional education and support regarding the LCS landscape in order to achieve informed decision making.

## Graphical abstract

We implemented a survey that included a full‐profile conjoint valuation analysis (CVA) survey using a nationally representative web‐based panel. Overall propensity for screening is low in a high risk population, and that out of pocket costs were of greater importance to potential screeners than mortality reduction or false positive rates.

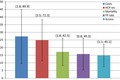

## 1 INTRODUCTION

Lung cancer is the leading cause of cancer mortality in the United States, with an estimated 228 150 new cases and 142 670 estimated death for 2019.^1^ In 2011, the National Lung Cancer Screening Trial reported a 20% relative reduction in lung cancer mortality with annual low‐dose computed tomography (LDCT) compared to traditional chest X‐ray.^2^ Subsequently, the US Preventive Services Task Force gave a Grade B recommendation for lung cancer screening (LCS) using LDCT for high‐risk individuals,^3^ prompting comparable organizational guidelines.^4^, ^5^, ^6^, ^7^, ^8^, ^9^ Additional data emerging from the NELSON trial similarly support the decision to disseminate LCS to high‐risk individuals.^10^ All LCS recommendations emphasize informed and shared decision making.^11^

The inclusion of shared decision‐making in LCS guidelines reflects the fact that LCS generates notable potential harms as well as benefits.^12^ The principal harms include false‐positive results,^2^ over‐diagnosis,^13^, ^14^ radiation exposure,^15^ and psychological distress, specifically for patients who receive an indeterminate or positive result.^16^, ^17^ Additional harms, such as financial strain and opportunity costs, have also been noted.^18^ An understanding of the balance between the benefits and potential harms of LCS is necessary for patients to formulate preferences and make informed decisions.

The current literature for LCS demonstrates that while patient interest in LCS is generally high, preferences are influenced by a number of patient and screen‐specific factors.^19^, ^20^, ^21^, ^22^ One unique approach to LCS preference elicitation involves conjoint valuation analysis (CVA), which measures the joint effects of two or more independent characteristics on an individual's appraisal of a service/product. Through evaluation of a series of hypothetic scenarios, the relative importance of the different attributes that affect an individual's choice is elicited. Unlike a direct rating of the importance of each attribute in isolation, conjoint analysis forces trade‐offs in the importance of the different attributes.^23^ Although the conjoint methodology approach to preference elicitation in cancer screening has been used increasingly by decision‐making researchers [eg, 24, 25, 26, 27, 28], it has not been employed to study LCS preferences.

The objectives of this research were to: (a) better understand how individuals who are at higher risk of lung cancer view and value the different characteristics of LCS procedures; (b) evaluate the overall propensity for LCS adoption; and (c) explore individual characteristics that correlate with attribute preferences and overall screening propensity.

## 2 MATERIALS AND METHODS

### 2.1 Overview

This study was approved by the University of Miami and University of Kentucky Institutional Review Boards. We conducted an online survey of a nationally representative sample (n = 210) of individuals at increased risk for lung cancer. The survey collected information on respondent demographic characteristics, and included a conjoint exercise to assess respondents' attitudes toward different aspects of LCS. Respondents were identified and the survey administered using an internet‐based survey panel, KnowledgePanel® (now GfK Knowledge Networks; http://www.knowledgenetworks.com/GANP/).

### 2.2 Respondents

Knowledge Networks^TM^ (KN) conducted online surveys using the web‐enabled KnowledgePanel®, a probability‐based panel designed to be representative of the United States population.^29^ To establish and maintain its panel, KN conducted random digit dialing. Persons in selected households were invited to participate in the panel and provided with an internet appliance and connection, if needed.

The target population included English‐speaking individuals 45 years of age or older who were former or current smokers with at least a 20 pack‐year history and had no history of lung cancer. This sample was selected as representing individuals who are at higher risk for lung cancer, and who maybe be in a position to consider screening (even if they do not meet current criteria). Additionally, we targeted a sample that was approximately 50% female; 25% African American and 25% Hispanic; and 25% rural dwelling. The target sample size of n = 200 was based on a conservative approach to the sample size estimation algorithm for conjoint methodology.^30^ The available background information on KN Panel members included smoking history and current smoking status, but not enough information to calculate pack‐years smoked. Therefore, initial eligibility questions for KN panel participants were needed to establish individuals' overall tobacco exposure. Of the 525 individuals invited to participate, 223 met eligibility criteria including smoking history, and 210 individuals completed the online survey (94%).

### 2.3 Survey development

The survey included standard demographic and smoking related items drawn from previous federal surveys. However, the CVA instrument was newly developed as part of this study. Conjoint methodologies were originally developed in psychology and most broadly employed by marketing researchers. More recently, conjoint methods have been increasingly been used in medical decision‐making context to assess the importance of factors that influence decisions about healthcare services.^31^, ^32^, ^33^

**Figure 1:**
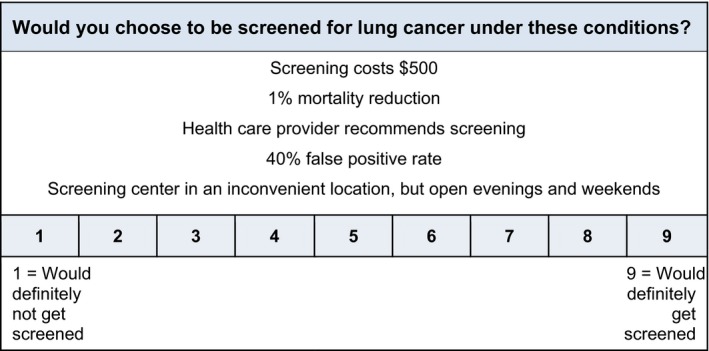
Example lung cancer screening scenario used in the CVA

In developing a conjoint evaluation instrument, we conducted semi‐structured interviews with 40 smokers and 9 health‐care providers to collect information on LCS characteristics that might be most important and salient regarding individuals' decisions about screening. Based on these interviews and team expertise, we identified five attributes of LCS that were likely to be influential in decisions about screening. These attributes, along with the different levels, are listed in Table 1. Aiming for orthogonality and parsimony in our selection of scenarios, we used Sawtooth software (Inc Orem, UT) to identify 20 unique conjoint scenarios. Survey respondents rated each scenario on a 9‐point Likert type ratings scale anchored by “would definitely not get screened” and “would definitely get screened.” See Figure 1 for an example scenario.

**Table 1:** Lung cancer screening characteristics/attributes explored in the study

Characteristics/Attributes	Levels
Out of pocket costs	$100 out of pocket cost $300 out of pocket cost $500 out of pocket cost
Mortality reduction	A 1% reduction in lung cancer deaths A 10% reduction in lung cancer deaths A 20% reduction in lung cancer deaths
Health‐care provider recommendation	Your doctor recommends that you do not get screened Your doctor recommends that you do get screened Your doctor says that you should make the decision You do not discuss screening with your doctor
False‐positive rate	10% false‐positive rate 25% false‐positive rate 40% false‐positive rate
Ease of access	Imaging center is in a convenient location and is open in evenings and weekends Imaging center is in a convenient location but is open from 9 am to 5 pm on weekdays only Imaging center is in an inconvenient location but is open in evenings and weekends Imaging center is in an inconvenient location and is open from 9 am to 5 pm on weekdays only

### 2.4 Online survey procedures

Knowledge Networks^TM^ sent invitations in batches to panel members likely to meet the eligibility criteria. As mentioned above, the KN databases did not include sufficient information to calculate volume of tobacco exposure, so additional assessment of pack‐years was necessary to determine eligibility. Each respondent received a $30 honorarium. All data were entered directly into online databases so there were few missing data. Data were only missing if a participant refused to answer questions or terminated the survey before it was completed.

### 2.5 Data analysis

Data analysis included both descriptive summaries of data and multivariate statistics. All analyses were performed using Stata 13.0 and/or Sawtooth Software. Summary statistics for all demographic characteristics were calculated first.

Using Sawtooth software, we calculated relative importance scores for attributes and part‐worth utilities for each respondent, as well as the mean for the sample population. Relative importance of an attribute in our analyses is a measure of how much influence each characteristic (such as cost or false‐positive rate) has on an individual's willingness to be screened. Relative importance scores for each individual sum to 100. Part‐worth utilities reflect the desirability (or lack of undesirability) of a given level of an attribute. We used a hierarchical Bayes approach,^34^ which uses averages (information about the distribution of utilities from all respondents) as part of the procedure to estimate attribute level utilities for each individual.

To characterize each respondent's overall propensity for screening, we calculated the average of their ratings (scale of 1‐9) for the 22 conjoint scenarios.

Finally, we used univariate regression analyses to examine associations between demographic characteristics (all characteristic in Table 2) and (a) the relative importance ratings for each attribute and (b) the average propensity to screen. We used a Bonferroni correction to ameliorate the chance of Type 1 errors due to multiple comparisons.

**Table 2:** Study participants' sociodemographic characteristics (n = 210)

	% (n)
Age (mean ± SD, yrs)	60.69 ± 8.46
Female	51.90 (109)
Current smoker	40.58 (84)
Pack years smoking (mean ± SD)	39.95 ± 20.10
Race/ethnicity	
White, non‐Hispanic	46.38 (96)
Black, non‐Hispanic	25.12 (52)
Hispanic	28.50 (59)
General health status	
Excellent	5.77 (12)
Very good	21.63 (45)
Good	46.15 (96)
Fair	22.60 (47)
Poor	3.85 (8)
Education	
Less than high school	12.86 (27)
High school education	32.86 (69)
Some college	37.62 (79)
Bachelor's degree or higher	16.67 (35)
Marital status	
Partnered	66.2 (139)
Single	33.8 (71)
Income	
Less than $15 000	15.24 (32)
$15‐25 000	11.90 (25)
$25‐35 000	10.48 (22)
$35‐50 000	15.71 (33)
$50‐75 000	20.00 (42)
Over $75 000	26.67 (56)

## 3 RESULTS

### 3.1 Characteristics of study respondents

Mean age of respondents was 61 years (SD 8.5), and 51.9% of respondents were female (Table 2). Average pack‐years of smoking was 40 (SD 20.1), with early onset of both first smoking (16 years, SD 2.9) and regular smoking (18 years, SD 5.3). Less than half of respondents were current smokers (40.6%) and, of those, 74.7% had tried quitting. The majority was married or cohabitating (66.2%). Consistent with our desired sampling frame, respondents were 46.4% White, non‐Hispanic; 25.2% Black, non‐Hispanic; and 28.50% Hispanic. Respondents were relatively evenly distributed across income groups, and most had a high school education or some college (70.5%). About half of respondents had medical insurance through an employer. Of the 210 participants, 74.6% reported being in “good” to “excellent” health.

### 3.2 Relative importance of the attributes of the lung cancer screening scenarios

**Figure 2:**
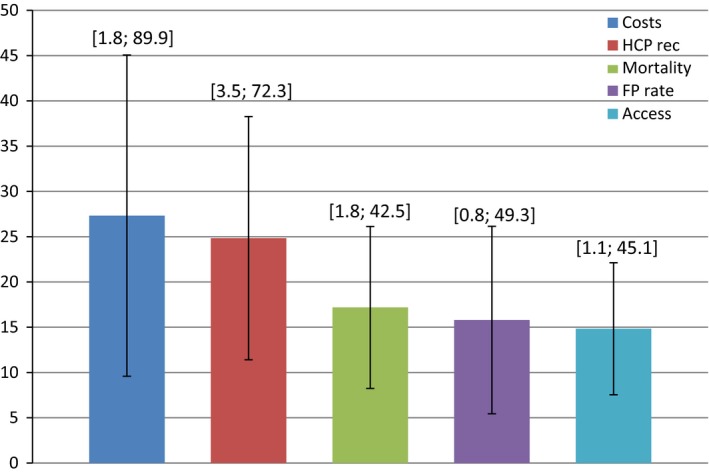
Mean (SD) importance scores for conjoint scenario attributes; minimum and maximum individual values in brackets

As described, the relative importance of an attribute is a measure of how much influence each attribute has on respondent choices. The average relative importance of the five attributes varied from a high of 27.3 (SD 17.7) for out of pocket costs to a low of 14.8 (SD 7.3) for ease of access (Figure 2), indicating that cost is likely to play a highly influential role in screening implementation. However, all of the attributes appeared to influence choices, including provider recommendation (24.8, SD 13.4), mortality reduction (17.2, SD 8.9), and false‐positive rate (15.8, SD 10.4).

Variation in individuals' relative importance scores and propensity for screening was quite large (Figure 2). Individual importance scores ranged from the single digits (for all five attributes) to over 50 (for health‐care provider recommendation – max of 64% and out of pocket cost – max of 87%). Similarly, average propensity to be screened also varied widely from 1 to 9.

### 3.3 Part‐worth utilities for attribute levels

**Figure 3:**
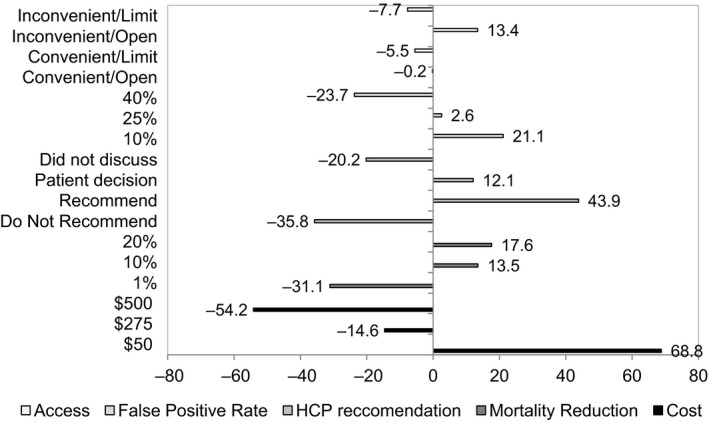
Attribute part‐worth utilities from CVA

Part‐worth utilities measure the “desirability” of each level of an attribute relative to the other levels of that attribute. For example, the attribute of false‐positive rate has part‐worth utility scores of: 21.1 for a 10% false‐positive rate, 2.6 for a 25% false‐positive rate, and −23.7 for a 40% false‐positive rate (Figure 3). This indicates that respondents find a 10% false‐positive rate much more desirable than a 40% false‐positive rate. The part‐worth utility values for levels in a given attribute are zero‐centered, and sum to zero.

Patterns within the part‐worth utility values for the levels of out of pocket cost, mortality reduction, and false‐positive rate are consistent with the natural ordering, as expected. $50 out of pocket costs was preferred to $500 (68.8 vs −54.2), 20% mortality reduction was preferred to 1% (17.6 vs −31.1), and 10% false‐positive rate was preferred to 40% (21.1 vs −23.7). In addition, a health‐care provider recommendation against LCS had the lowest part‐worth utility (−35.8) for that attribute, indicating that screening was least desirable when a provider recommended against it. In terms of access, respondents found the LCS conditions with extended hours most desirable.

### 3.4 Propensity to be screened

The average propensity for screening, on a scale of 1‐9, was just 3.63 (SD 1.6), indicating relatively low inclination for screening. Among the 22 hypothetical scenarios, the average ratings ranged from 2.60 (SD 2.00) to 5.57 (SD 2.13). The most favorable screening scenario's attributes were: $100 out of pocket costs; 10% reduction in mortality; PCP recommendation to screen; 10% false‐positive rate and inconvenient location but open evenings and weekends. Even with these characteristics, the average willingness to be screened was only slightly over the midpoint from "definitely no" to "definitely yes". (NB: Since not all combinations of attribute levels were used for conjoint scenarios, our "best" scenario in the survey was not the "best possible" scenario.)

### 3.5 Univariate associations

After planned Bonferroni adjustment for multiple comparisons, the only significant associations of important scores/propensity to be screened with respondent demographic characteristics were: (a) a negative association between income and the importance of cost for screening decisions (*P* < 0.05), and (b) a positive association between race/ethnicity of Black non‐Hispanic and higher average propensity to be screened (*P* < 0.05).

## 4 DISCUSSION

Low‐dose CT screening has been shown to reduce lung cancer mortality in selected high‐risk groups and is covered by both Medicare and a large number of private insurance companies. However, screening is not without risks and constitutes a quintessential “preference‐sensitive” decision. Therefore, it is important that informed decision making for LCS be emphasized, and as part of that, understanding the factors that affect higher risk individuals' attitudes toward screening and screening decisions provides key information on how to engage candidates. This research provides unique information on attributes of LCS and the influence these aspects likely have on decisions to pursue screening.

Results show the relative importance values that potential screening candidates place on 5 screening attributes previously identified as potentially influential through qualitative research (see Table 1). Specifically, decisions about screening were most highly influenced by cost, with health‐care provider recommendations a close second. Surprisingly, reduction in mortality and false‐positive rates associated with screening was not nearly as influential on decisions at a population level. However, we also found a wide variation in which factors/attributes were most influential.

Similar to other cancer screening contexts, we found that clinician recommendations played a substantial role in patient ratings of anticipated screening [eg, 35], highlighting the importance of clinician‐patient communication and training with regard to LCS and shared decision making. However, some previous studies of primary care clinicians^36^, ^37^, ^38^, ^39^ have demonstrated suboptimal knowledge, attitudes, and practices with regarding to LCS. For example, only 22%^36^ to 31%^39^ of primary care providers knew the correct LCS eligibility criteria, which led to inappropriate LCS referrals. Thus, our research highlights the need for up‐to‐date knowledge about LCS among primary care providers.

Previous research on LCS has shown relatively favorable attitudes toward screening when participants are asked about interest in screening or willingness to be screened [eg, 19, 20, 21, 22]. However, conjoint‐generated "propensity" to be screened results show generally low levels of interest in screening when the respondent is considering a more fully described screening scenario. These findings are in alignment with LDCT screening uptake data, specifically that the actual number of people being screened for lung cancer following publication of the NLST is lower than anticipated.^40^, ^41^

Achieving optimal implementation of LCS (including individual preferences as well as objective risk factors) requires development of comprehensive and organized LCS programs and rigorously quality standards. To date, this has not occurred on a national level (only individual programs). Part of the reason for this may be the relative recency of recommendations for LCS by USPSTF and other organizations. However, other barriers to achieve optimal screening—which may be even more difficult to overcome include achieving suitable availability and access to high‐quality screening programs, and improving patient navigation across the screening cascade.

Limitations of this study are consistent with other studies that elicit preferences and explore decision making using conjoint methods. First, attribute preferences and intention to screen in a hypothetical scenario are treated as an indication of real‐world attribute preferences and actual screening behavior, despite their differences.^42^ Second, the attribute information is, by necessity, presented in a simplified way which may lead to biases in judgement. Finally, despite a degree of oversimplified attribute information, conjoint analyses can be complex for participants. However, we restricted our conjoint scenarios to a number previously shown not to induce decision fatigue,^43^ and the order of scenario presentation was randomized to reduce potential order effects.

Our study also has several important strengthens. Our sample population was nationally representative with additional oversampling by race/ethnicity. Our conjoint scenarios were developed using a rigorous mixed methods approach. Use of the conjoint methodology allowed us to capture a variety of benefits, harms and other potentially important factors in LCS decisions. Finally, qualitative feedback indicated great potential for the future use of conjoint exercises as part of an informed and shared decision making process for LCS.

## 5 CONCLUSIONS

Overall, findings highlight the need for patients to be educated about both the benefits (eg, mortality reduction) and risks (eg, false‐positive rates) of LCS. Information on these and other aspects of screening has to be presented in a way that is comprehensible and relevant. In addition, if we want to help individual patients make the most appropriate decision about screening for themselves, eliciting preferences and putting data in the context of the screening candidate must be a vital component of that process. Only in this way will we be able to ensure quality decision making for LCS.

## CONFLICT OF INTEREST

No authors have any conflicts of interest to report.

## AUTHOR CONTRIBUTIONS

All listed authors meet the four criteria definition of authorship: (a) Substantial contribution to the conception, design, analysis, or interpretation; (b) Contributed to drafting or revising the manuscript; (c) Provided final approval of the submitted version; and (d) Agree to be accountable for all aspects of the work.

MMB had full access to all of the data in the study and takes responsibility for the integrity of the data and the accuracy of the data analysis, including and especially any adverse effects. MMB, RJT, and JLS contributed substantially to the study design, data analysis and interpretation, and the writing of the manuscript.
